# Neuroinvasive West Nile virus infections after solid organ transplantation: Single center experience and systematic review

**DOI:** 10.1111/tid.13929

**Published:** 2022-08-30

**Authors:** Anum Abbas, Fang Qiu, Adia Sikyta, Paul D. Fey, Diana F Florescu

**Affiliations:** ^1^ Infectious Diseases Division University of Nebraska Medical Center Omaha Nebraska USA; ^2^ Department of Biostatistics, College of Public Health University of Nebraska Medical Center Omaha Nebraska USA; ^3^ Department of Pathology and Microbiology University of Nebraska Medical Center Omaha Nebraska USA

**Keywords:** heart transplantation, intestinal transplantation, kidney transplantation, liver transplantation, lung transplantation, neuroinvasive, pancreas transplantation, solid organ transplantation, West Nile virus

## Abstract

**Abstract:**

West Nile virus (WNv) is a major cause of viral encephalitis in the United States. WNv infection is usually asymptomatic or a limited febrile illness in the immunocompetent hosts, although a small percentage can develop neuroinvasive disease. Neuroinvasive disease due to WNv in solid organ transplant recipients occurs at higher rates than observed in the general population and can have long term neurological sequalae.

**Methods:**

We retrospectively reviewed medical records of all solid organ transplant recipients at our institution who tested positive for WNv from 2010 to 2018. Two reviewers performed electronic searches of Medline, Embase, Cochrane Library of literature of WNv infections in SOT. Descriptive statistics were performed on key variables.

**Results:**

Eight recipients (mean age 54, five males) were diagnosed with neuroinvasive WNv infection at our institution. Distribution of infection was as follows: five kidney transplants, one in each kidney‐pancreas, liver, and lung. Diagnoses included meningitis (3), encephalitis (1), meningo‐encephalitis (4). Median time from transplant to infection was 49.8 months (2.7–175.4). No infections were considered donor‐derived. Five patients received treatment with IVIG. Six patients were alive at median follow‐up of 49.5 months (21.7–116.8). We identified 29 studies published from 2002 to 2019. Median time from transplant to infection was 14.2 months, with similar allograft distribution; 53% were donor‐derived infections.

**Conclusion:**

WNv infections in solid organ transplant recipients can be a consequence of organ donation or can be acquired via the community. Infections can be more severe in SOT recipients and lead to neuroinvasive disease.

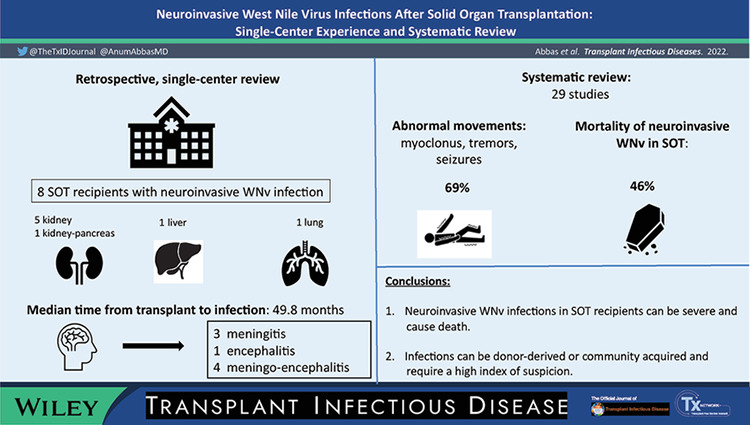

## 1 INTRODUCTION

West Nile virus (WNv), a positive single‐stranded RNA virus, is the most common cause of epidemic viral encephalitis in the United States. The majority of WNv infections are related to infected mosquito bites; blood transfusions, solid organ transplant (SOT), breast milk, trans‐placental, and percutaneous injuries are less common modes of transmission.^1^ WNv infection mostly presents as a nonspecific febrile illness or is asymptomatic, but neuroinvasive disease can develop in <1% of those infected.^2^, ^3^ In more than two decades since the first detection of WNv infections in United States, over 25,000 neuroinvasive cases have been reported.^4^ SOT recipients are at higher risk of developing neuroinvasive disease with WNv infections and can acquire infection via organ transplantation. Several reviews of donor‐derived WNv infection report a high incidence: 87% of the recipients of an organ from WNv positive donor were diagnosed with infection; 75% of the infected recipients had neuroinvasive disease and 25% remained asymptomatic; 30% of recipients died or had severe neurological impairment^5^, ^6^ .

We describe experience of WNv infections in SOT recipients at a large academic transplant center and provide a systematic review of the literature.

## 2 METHODS

**Patient selection**. We retrospectively reviewed the electronic medical records of all adult SOT recipients at our institution who tested positive for WNv in blood and/or CSF from January 2010 through December 2018. Collected data included: patient demographics; allograft type; date of transplant; type of induction and maintenance immunosuppression; length of hospital and ICU (intensive care unit) stay; type of WNv infection (meningitis, encephalitis, meningoencephalitis, acute flaccid paralysis); clinical presentation; abnormal movements; treatment (IVIg, interferon, reduction in immunosuppression); donor derived infections; graft and survival outcomes.

**Literature Search. Data Extraction**. PubMed, the Cochrane Library databases, and EMBASE were searched from inception to February 2020. A combination of keyword‐ and subject heading‐based search strategies was used in all databases and included WNv, transplant, organ transplant, organ transplantation, SOT, liver transplant/transplantation, lung transplant/transplantation, heart transplant/transplantation, small bowel transplant/transplantation, intestinal transplant/transplantation, kidney transplant/transplantation, pancreas transplantation/transplantation, and donor derived infection. Only articles in English and French were included. Two authors performed the literature search. Twenty‐nine articles were retrieved by the searches. Two authors performed the study selection independently. Any disagreement was resolved by discussion between the two authors. The PRISMA criteria were used for the search and flow of studies. Inclusion criteria: adult SOT recipients diagnosed with neuroinvasive WNv infection. Exclusion criteria: pediatric SOT patients; WNv infection prior to transplantation; if >2 articles reported the same patient/patients, we included the article with more data points; we excluded articles in which demographics were not described to avoid duplicating data; WNv diagnosed postmortem.

The following variables were collected from all studies: authors, publication year; demographics (sex, age); comorbidities (diabetes, hypertension, chronic kidney failure, cardiovascular and liver disease); allograft type; induction, and maintenance immunosuppressive therapy; time to infection after transplant; clinical presentation (type of neuroinvasive disease, initial symptoms, presence of abnormal movements); serologic and NAT (nucleic acid test) diagnosis (serum and cerebrospinal fluid); time to establishing the diagnosis; EEG (electroencephalogram) results; treatment (intravenous immunoglobulin, interferon), neurological sequelae (at one year and long term); outcomes (graft loss, death, attributable mortality); if donor‐derived infection.

**Definitions**. WNv encephalitis ‐ presence of altered mental status or acute personality changes in combination with at least two of the following: focal neurologic deficits, seizures, leukocytosis, CSF (cerebrospinal fluid) pleocytosis, positive WNv IgM in the serum or CSF, positive WNv NAT in the blood or CSF, EEG findings consistent with encephalitis and abnormal neurological findings consistent with WNv. Meningitis ‐ signs of meningeal inflammation, photophobia associated with one of the following: leukocytosis, CSF pleocytosis, positive WNv IgM in the serum or CSF, positive WNv NAT in the blood or CSF, neuroimaging demonstrating meningeal inflammation. Acute flaccid paralysis: acute progressive weakness of extremities associated with 2 of the following: weakness asymmetry, areflexia or hyporeflexia, high CSF protein level, CSF pleocytosis, positive WNv IgM in the blood or CSF, positive WNv NAT in the blood or CSF, and abnormal signal in anterior gray matter on imaging. Donor‐derived infections are defined as any infection present in the donor that is transmitted to one or more recipient.^7^

The donors were screened according as per each Organ Procurement Organization policy. If the recipient was suspected of WNv infection, we sent WNv‐specific serology (IgG and IgM) and NAAT in the blood. If a lumbar puncture was performed, paired blood and CSF samples were sent for WNv‐specific serology (IgG and IgM) and NAAT.

**Statistical analysis**. Descriptive statistics were performed for all key variables included in this study. Overall survival from hospital admission was plotted using Kaplan–Meier method.

## 3 RESULTS

Eight SOT recipients with mean age of 54.47 years (standard deviation [SD] 12.79) were diagnosed with WNv infection at our institution during the study period; 62.5% were male (**Table** **1**). The infections were diagnosed in Agust (3), September (3), and October (2). All patients with WNv infection were hospitalized. Allograft distribution was as follows: five (62.5%) kidney, and one (12.5%) of each kidney, kidney‐pancreas and liver transplant recipients. Comorbidities included hypertension (100%), diabetes mellitus (87.5%), cardiovascular disease (62.5%), chronic kidney disease (37.5%), and liver disease (12.5%). Induction therapy: basiliximab (37.5%), thymoglobulin (37.5%), alemtuzumab (12.5%); one patient did not have induction therapy. Only one patient had rejection within 1 year of the infection and was treated with thymoglobulin. Maintenance immunosuppression at the time of WNv infection: 100% calcineurin inhibitors, 87.5% mycophenolate mofetil, and 62.5% steroids. The median time‐to‐WNv infection after transplant was 49.82 months (range 2.73–174). The mean time from hospital admission to confirmed diagnosis was 3.25 (SD 2.05) days. Mean hospital length of stay was 19.13 (SD 14.6) days; five (62.5%) patients were admitted to intensive care unit with a mean length of stay of 13.2 (SD 7.26) days. Immunosuppression was decreased in seven (87.5%) patients. Five (62.5%) patients were treated with intravenous immunoglobulin, with a mean total dose of 1340 mg/kg (SD 444.97). No patients received interferon. Six patients (75%) were alive at a median follow‐up of 49.5 (range 21.7–116.8) months. No abnormal movements were reported in our series, but five of eight patients had an abnormal EEG. Two patients had neurologic sequalae, one had foot drop, and the other had chronic dizziness. Two patients had death attributable to WNv neuroinvasive disease.

**TABLE 1:** Characteristics of SOT recipients with WNv infection (*n* = 8)

**Characteristics**	
Mean age in years (SD)	54.47 (12.79)
Male sex (%)	5 (62.5)
Allograft (%)	
Kidney	5 (62.5)
Kidney/pancreas	1 (12.5)
Liver	1 (12.5)
Lung	1 (12.5)
Comorbidities (%)	
Diabetes mellitus	7 (87.5)
Chronic kidney disease	3 (37.5)
Liver disease	1 (12.5)
Immunosuppression (%)	
CNI	8 (100)
MMF	7 (87.5)
Steroids	5 (62.5)
Median time to infection from transplant in months (range)	49.82 (2.73–173)
Treatment of rejection within 1 year of infection (%)	1 (12.5)
Treatment with IVIG (%)	5 (62.5)
Decreased immunosuppression (%)	7 (87.5)
Donor derived infection (%)	0 (0)

*Note*: CNI, calcineurin inhibitors; IVIG, intravenous immunoglobulin; MMF, mycophenolate mofetil; SD, standard deviation; WNv, West Nile virus.

Twenty‐nine‐published studies were included in the systematic review, and the results are presented in **Table** **2**. For the patients in the systematic review, the mean age was 50.2 years (SD 13.9), and 69.8% were male. Maintenance immunosuppression was as follows: 98.04% calcineurin inhibitors; 97.6% mycophenolate mofetil; 88.9% steroids and 8.57% sirolimus. Abnormal movements were reported in 69% of patients in the systematic review. These were reported as myoclonus, tremors, seizures, and nystagmus.

**TABLE 2:** Systematic review of WNv infection in SOT recipients

Variables		Number of patients for which data were available
Mean age in years (SD)	50.21 (13.99)	53
Male (%)	37 (69.81)	53
Allograft type (%) Kidney Kidney‐pancreas Pancreas Liver Heart Lung	30 (56.6) 5 (9.43) 1 (1.89) 7 (13.21) 6 (11.32) 4 (7.55)	53
On calcineurin inhibitors at the time of infection (%)	50 (98.04)	51
On sirolimus at the time of infection (%)	3 (8.57)	35
On MMF at the time of infection (%)	43 (87.76)	49
On steroids at the time of infection (%)	40 (88.89)	45
Median time‐to‐infection after transplant in months (range)	14.01 (0.1‐180.13)	48
Mean time‐to‐diagnosis in days (SD)	5.21 (6.67)	43
Classification of infections (%) Asymptomatic infection WNv fever Encephalitis Encephalitis, myelitis, and flaccid paralysis Flaccid paralysis Meningitis Meningoencephalitis Meningoencephalitis and flaccid paralysis	1 (1.89) 4 (7.55) 22 (41.51) 5 (9.44) 1 (1.89) 2 (3.78) 15 (28.30) 3 (5.66)	53
Donor derived infection (%)	13 (24.53)	53
Presence of abnormal movements (%)	29 (69.05)	42
Decreased immunosuppression (%)	35 (97.22)	36
Treatment with IVIG (%)	24 ( 45.2)	24
Treatment with interferon (%)	11 (68.75)	16
Long term neurological sequelae (%)	9 (18.75)	48
Graft loss (%)	2 (3.85)	52
Death (%)	19 (36.54)	52
Attributable mortality (%)	16 (51.61)	31

*Note*: MMF, mycophenolate mophetil; SD, standard deviation; WNv, West Nile virus.

## 4 DISCUSSION

This case series and systematic review of WNv neuroinvasive infections in SOT recipients demonstrates the potential severity of WNv disease in SOT recipients and highlights an underrecognized pathogen. We report eight cases of neuroinvasive WNv infection in SOT recipients at our institution over the study period. Our institution performs heart, lung, liver, kidney, pancreas, and small bowel transplants and performed an average of 137 transplants per year during the study period. We noted a preponderance of cases in kidney recipients (five of eight cases); kidney transplants are a more common procedure; these recipients are more likely to return to normalcy faster compared to other allograft recipients; also studies in nonhuman primates demonstrated that kidneys can be a reservoir of WNv, potential source for donor‐derived infections.^8^ No intestinal or multivisceral transplant recipients were reported to be infected with WNv, possibly secondary to longer hospitalizations after transplant and limited outdoor exposure. Time‐to infection after transplant was variable, shorter if the infection was blood or donor‐derived and longer if the infection was community acquired. In our study, the time‐to diagnosis of WNv infection was shorter, probably reflecting lessons learned over time. We also report that majority of cases were diagnosed as encephalitis and meningoencephalitis, while myelitis and flaccid paralysis were rare.

WNv can be transmitted via transplanted organs and blood transfusions.^9^, ^10^, ^11^ The first donor derived WNv infections were reported in 2003^9^ and since another 19 cases have been reported.^1^ Blood and transplant transmitted WNv infections significantly decreased after screening blood and organ donors, especially after introduction of more sensitive NAT tests.^3^ Testing donors for WNv infection is recommended when there is known active disease in the donor's region.^1^ However, a recent survey published by Theodoropoulos et al. showed that only 39% of the organ procurement organizations in the US were conducting WNv screening of donors.^12^ None of the cases in the current series were considered to be donor‐derived infections, most likely due to low number of cases included. Almost 25% of cases in the systematic review were classified as donor‐derived. During the study period of our series, the overall incidence of neuroinvasive disease in the state was higher; a total of 358 neuroinvasive cases reported in the state.^13^ Donor screening practices and having a high index of suspicion during mosquito active months have contributed to decreased transmission from donors and earlier detection in recipients.

The clinical presentation of WNv in SOT seems to be more severe than in general population with a higher likelihood of neurological involvement. Neuroinvasive disease, which is estimated to occur in <1% in the general population, is around 2.5% in solid organ transplant recipients with incidence up to 75% when infection is acquired via blood or organ donation.^1^ The initial clinical presentation can be nonspecific; therefore a high index of suspicion for WNv infections should be present during high mosquito activity period and in SOT recipients with fever and CNS (central nervous system) manifestations. The most common presentation for WNv infection is fever, followed by neurological manifestation (abnormal movements, weakness, mental status alteration) and gastrointestinal symptoms (nausea, vomiting, diarrhea, abdominal pain).^9^, ^10^, ^11^, ^14^, ^15^, ^16^, ^17^, ^18^, ^19^, ^20^, ^21^, ^22^, ^23^, ^24^, ^25^, ^26^

Neuroinvasive WNv infections, although diagnosed in a minority of patients, can lead to long‐term neurologic, neuropsychologic and cognitive impairment and is associated with increased mortality ^1^ and isolated acute flaccid paralysis.^27^ Older age (>60 years), male gender, diabetes, hypertension, chronic renal disease, history of cancer, and alcohol abuse are risk factors for severe disease and encephalitis, while hypertension was a risk factors for meningitis.^27^, ^28^ Immunosuppression was not a risk factor for severe illness or neuroinvasive disease but was associated with increased risk of death ^27^, ^29^, ^30^ ^.^ Most transplant recipients with WNv infections have been on MMF, calcineurin inhibitor and steroids regimen, that has an impact on both, B and T cells.^9^, ^10^, ^11^, ^14^, ^16^, ^18^, ^22^, ^23^, ^26^

Temporary reduction in immunosuppression is recommended for all patients to allow recovery of the immune system.^1^, ^21^ WNv, as with other flaviviruses, seems to be more susceptible to enhanced humoral immunity rather than cell mediated immunity, explaining why IVIG has been used to treat WNv infections. Cell mediated immunity also plays a role in control of infection by production of interferons/cytokines and limiting progression to CNS disease, thus, impaired cell mediated immunity in SOT recipients may limit ability to clear infection.^34^ There is no specific dose or length of treatment with IVIG recommended at this time and the benefit remains unclear.^10^, ^11^, ^14^, ^22^, ^23^, ^24^, ^26^ The titer of specific immunoglobulin in these studies has not been determined. Even more, immunoglobulins do not cross the blood‐brain barrier to alter the course of neurological disease.^24^, ^35^ Administration of IVIg within 8 days of symptoms was associated with better clinical outcomes, but a causal relationship could not be demonstrated.^21^ Interferon alph‐2b has been administered to SOT recipients with WNv infections variable success.^10^, ^19^, ^26^ Interferon alpha‐2b seems to inhibit growth of WNv in vitro and enhances the immune response by increasing cytokine gene expression and function of monocytes, NK, and cytotoxic T cells. Although data are lacking, there is a theoretical concern that treatment with interferon might increase risk of allograft rejection.^1^ In our series, no patients were on sirolimus, and only 8% in the systematic review received sirolimus immunosuppression. Sirolimus is not first line immunosuppression at most institutions, which may account for the decreased use observed in the current series and the review. However, known antiviral activity of sirolimus is notable,^36^ and it is possible its use may be associated with less severe WNv infections, although no associations can be made based on this data.

In previous studies, SOT status was not associated with increased risk for severe illness and neuroinvasive disease, but the studies were underpowered to detect such an association. A WNv seroprevalence study showed that SOT recipients have a 40 times higher risk than general population for neuroinvasive disease.^31^ Mortality from donor derived WNv seems to be high; a study of kidney transplant recipients reported 69% 1‐year survival with meningitis and encephalitis.^32^ Fatality‐to case ratio in neuroinvasive disease was reported at 2% for meningitis and 12% for encephalitis.^33^

Mortality due to WNv is very high in SOT recipients. Patients with WNv encephalitis and flaccid paralysis were reported to have more prolonged hospitalization (mean 8–25 days and 11–68 days) than patients with WNv meningitis or fever (mean 4–12 days and 7 days).^28^ Mortality for WNv fever and meningitis was <2% in comparison with up to 46% for WNv encephalitis and reaching 100% in combination with flaccid paralysis.^28^ Altered mental status was reported to be a predictor factor of mortality. We had two attributable deaths to WNv neuroinvasive disease in our series.

## 5 CONCLUSIONS

Our case series and review of literature demonstrate the devastating effects of WNv infections in SOT. The immunosuppression required to prevent rejection increases the risk of severe illness, in particular neuroinvasive disease. Flaccid paralysis seems to be associated with the highest mortality, followed by encephalitis. Physicians should have a high index of suspicion for neuroinvasive WNv infection in SOT recipients presenting with fever and neurological abnormalities, during the season when the mosquitos are active. Immunosuppression should be decreased as soon as possible.

## AUTHOR CONTRIBUTIONS

Conceptualization: DF. Writing: AA and DF. Review and editing: AA, PF, and DF. Biostatistics: FQ. Chart review, literature search, and study selection: AS and DF. All authors have read and agreed to the published version of the manuscript.

## CONFLICT OF INTEREST

The authors declare no conflict of interest.

## FUNDING INFORMATION

The authors received no specific funding for this work.

## Supporting information

Visual Abstract
