# Supplementary material for: Neuroinvasive West Nile virus infections after solid organ transplantation: Single center experience and systematic review
Source: Transpl Infect Dis. 2022 Aug 30;24(6):e13929. doi: 10.1111/tid.13929 (PMC10078393; doi:10.1111/tid.13929)
Supplement: Supplementary file 1 — Visual Abstract [file TID-24-0-s001.pptx]

## Slide 1
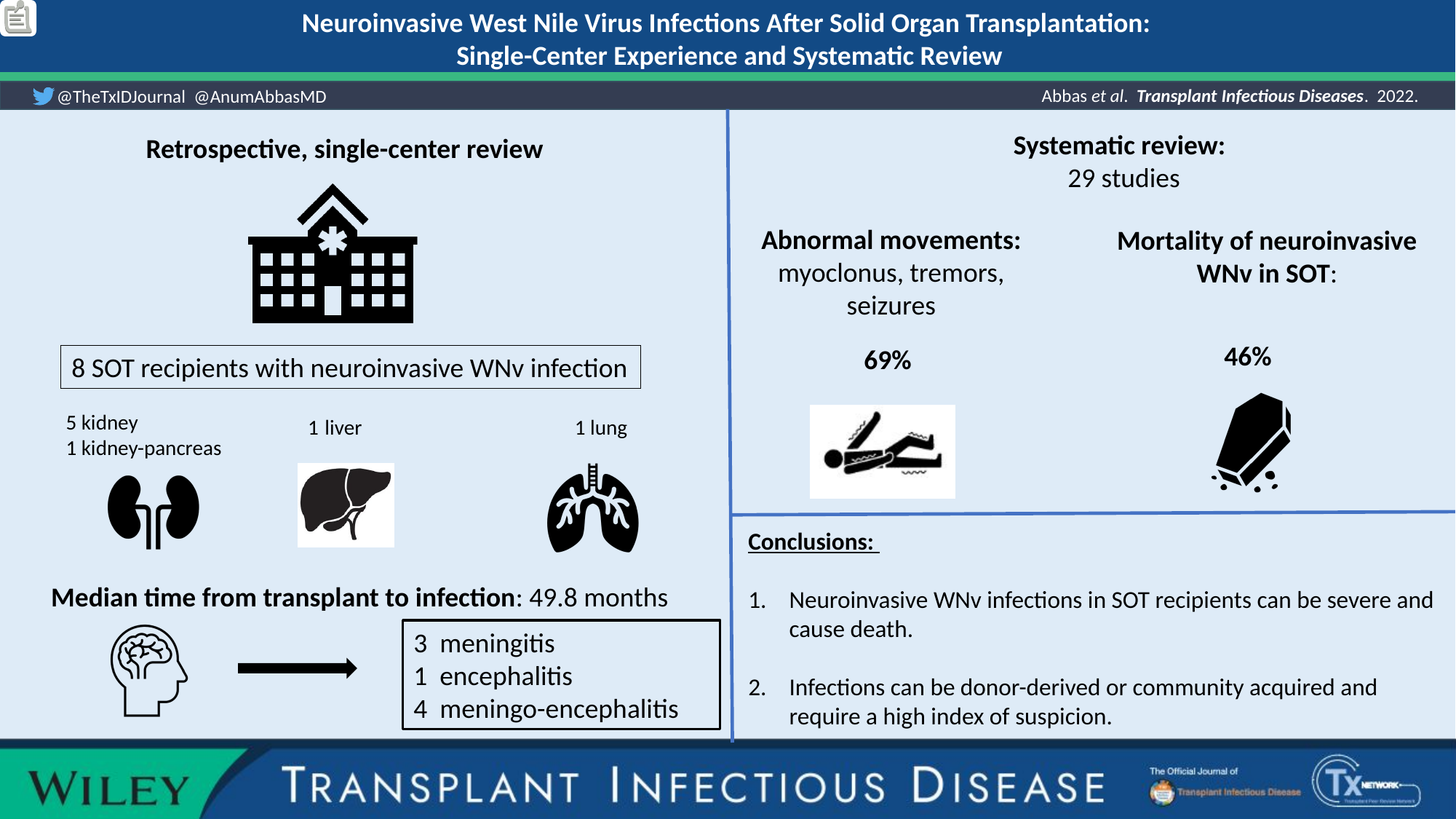

Neuroinvasive West Nile Virus Infections After Solid Organ Transplantation:
 Single-Center Experience and Systematic Review
Abbas et al. Transplant Infectious Diseases. 2022.
 @TheTxIDJournal @AnumAbbasMD
Systematic review:
29 studies
Retrospective, single-center review
Abnormal movements: myoclonus, tremors, seizures
Mortality of neuroinvasive WNv in SOT:
46%
69%
8 SOT recipients with neuroinvasive WNv infection
1 liver
5 kidney
1 kidney-pancreas
1 lung
Conclusions:
Neuroinvasive WNv infections in SOT recipients can be severe and cause death.
Infections can be donor-derived or community acquired and require a high index of suspicion.
Median time from transplant to infection: 49.8 months
3 meningitis
1 encephalitis
4 meningo-encephalitis
